# Effects of Rhizome Length and Planting Depth on the Emergence and Growth of *Alepidea amatymbica* Eckl. & Zeyh

**DOI:** 10.3390/plants9060732

**Published:** 2020-06-10

**Authors:** Ramatsobane Maureen Mangoale, Anthony Jide Afolayan

**Affiliations:** Medicinal Plants and Economic Development (MPED) Research Centre, Department of Botany, University of Fort Hare, Alice 5700, South Africa; maureem1@gmail.com

**Keywords:** *Alepidea amatymbica*, planting depths, rhizome length, cultivation

## Abstract

*Alepidea amatymbica* is used as a herbal medicine for the treatment of various diseases. As a result of its high medicinal value, this plant is being overexploited by herbal traders with little attention being paid to its conservation, which could lead to its extinction. Cultivation of *Alepidea amatymbica* was conducted to determine the appropriate planting depth and rhizome fragment length for the growth of this plant. The experiment was laid out in a Complete Randomized Block Design (CRBD) with two factors in a 6 × 3 factorial design. There were six levels of fragment length (1, 2, 3, 4, 5 and 6 cm) and three levels of burial depth (2.5, 5 and 7.5 cm). Emergence rate, number of leaves, leaf area, and plant height, number of florets, rhizome length gain, rhizome weight gain, shoot moisture, and rhizome moisture were measured as growth parameters. The best overall yield in terms of plant height, shoot emergence, rhizome weight gain, number of florets and number of leaves was observed in 7.5 cm planting depth at 6 cm rhizome length. Four- centimetre rhizome length had the highest leaf area of 111.9 ± 3.5 cm^2^, 101.3 ± 3.5 cm^2^, 105 ± 3.5 cm^2^ at 2.5, 5, 7.5 cm planting depth respectively. Shorter fragment lengths showed high potential for vegetative propagation in terms of rhizome length gain at all burial depths. These results suggest that *A. amatymbica* can regenerate from buried rhizomes and they may contribute to the establishment of a protocol for propagation that could help in conservation of this plant to avoid its extinction

## 1. Introduction

The intensive harvesting of wild medicinal plants has resulted in over-exploitation, which is a primary threat to biodiversity [1]. Over-exploitation of wild medicinal plants is mainly for commercial trade, which has become a form of self-employment in many rural areas [2]. Rock et al. [3] reported that medicinal plant species where underground parts are used are being exploited in natural habitats. *Alepidea amatymbica* is one such plant species that is heavily harvested and threatened by trade [4]. *A. amatymbica* is widely distributed in the grassland of the Eastern Cape, KwaZulu-Natal and Mpumalanga Provinces of South Africa and other Southern African Development Community (SADC) countries, such as Swaziland, Lesotho and Zimbabwe. The plant generally grows in riparian zones, along drainage lines, and forest margins at about 850–2500 mm above sea level [5].

*Alepidea amatymbica* has a long history of traditional use for the treatment of conditions such as asthma, cold, cough, influenza, sore throat, chest pains, headache, diarrhea, wounds, rheumatism and stomach ache [6,7,8]. In Zimbabwe, *A. amatymbica* is considered the tenth most recognised medicinal plant and is usually used as remedy for asthma and cough. The extracts of *A. amatymbica* have also been reported to be active against Human Immunodeficiency Virus (HIV) related diseases [9]. Important Kaurene type diterpenoids and derivatives ent-9, (11)-dehydro-16 Kauren-19-oic acid are known to constitute up to 11.8% of *A. amatymbica* rhizome and root dry mass [10].

The high demand for *A. amatymbica* rhizome by traditional healers and herbalists has caused extensive harvesting, which has resulted in the scarcity of the plant [11,12] According to Mander [13], *A. amatymbica* was ranked as the second most frequently traded herbal plant and it is in demand in the KwaZulu-Natal province. Dold and Cocks [11] reported that *A. amatymbica* is regarded as the most prevalent plant in Eastern Cape markets and estimated that more than 1200 tonnes is sold annually in the region. Due to its popularity in the markets, *A. amatymbica* harvesters travel to distant locations in the wild searching for it so that it can be available to consumers [14]. *Alepidea amatymbica* is being imported from Mozambique, Botswana, Zimbabwe, and Lesotho to South Africa, where it is scarce [15]. According to the South African Red List of Plants [16], there has been a drastic decline in the numbers of *A. amatymbica*. The population is estimated to have declined by at least 30% over the last three generations due to persistent harvesting pressure for the medicinal trade and some loss of suitable habitat for afforestation and cultivation [17]. O’Connor [18] also reported that *A. amatymbica* could be very common. However, its distribution has reduced due to overharvesting impacts on the plant. Due to the threat to its survival, *A. amatymbica* is of conservation concern in South Africa. One of the potentially effective measures for stemming over-exploitation of this plant is to encourage its propagation and cultivation.

The successful cultivation of medicinal plants is determined to a large extent by germination ability of the seeds [19]. However, the seeds of *A. amatymbica* have morphophysiological dormancy, which makes the germination of seeds difficult unless physiological dormancy is broken [20]. According to Mulaudzi et al. [21], temperature, light, cold scarification, smoke solution, and chemical substances can break the physiological dormancy of *A. amatymbica* after storage for three years. Although, seeds has been used for cultivation of *A. amatymbica*, seedling production using rhizome explants has never been done. Seedling production through micropropagation of rhizomes as explants and at appropriate planting depth has been reported by many researchers for *Laccosperma secundiflorum* [22], *Miscathus giganteus* [23] and *Tussilago farfara* [24] to improve cultivation.

Planting depth of rhizomes is an important factor in crop propagation practices because it influences the time of shoot emergence, vigour, and establishment [25]. Rhizome length and depth were found to influence the establishment of *M. giganteus* [23,26] also reported that burial depth of rhizomes was an important factor in determining yields in this species. Burial of the rhizome may greatly affect the survival and growth of clonal fragments because it can change biotic and abiotic conditions. The survival and growth of clonal fragments buried in deeper soils may mostly rely on the utilisation of reserves stored in plant organs when carbohydrates cannot be provided through photosynthesis [27]. However, if reserves stored in the plant organs are depleted before new shoots emerge from the rhizome, fragments are at risk of dying [28]. Successful cultivation of *A. amatymbica* would reduce harvesting pressure from natural populations, and it could help in the conservation of the plant, especially in the context of continued and possibly increasing demand. This study was therefore conducted to determine the appropriate rhizome length and planting depth suitable for the cultivation of *Alepidea amatymbica*, to encourage conservation of the species.

## 2. Materials and Methods

### 2.1. Plant Collection and Preparation of Planting Bags

Mature plants of *A. amatymbica* were collected in May 2014 from Emnyameni Village (32°34′17.39″ S, 27°6′55.16″ E) in the Keiskammahoek area of the Eastern Cape Province in South Africa. The area is classified as the Amathole Montana grassland [29] natural habitat. The plants were harvested with their rhizomes using a shovel to initially loosen the soil, and the dirt was removed around the rhizome. Plant materials were transported in polyethylene bags to Fort Cox College of Agriculture and Forestry greenhouse where the experiment was conducted. Plants were authenticated at the Department of Botany, University of Fort Hare, and voucher specimen Mau2015/04 were deposited at the Giffen Herbarium of the University. Top soil (0–15 cm) was collected in polyethylene bags and spread in the drying room for 48 h after which it was sieved through a 2 mm wire mesh. Five kilograms of soil was combined with pine bark (5 kg) to use as a growth medium in plastic bags of 14 cm diameter.

### 2.2. Preparation of Rhizome Fragments

**Figure 1:**
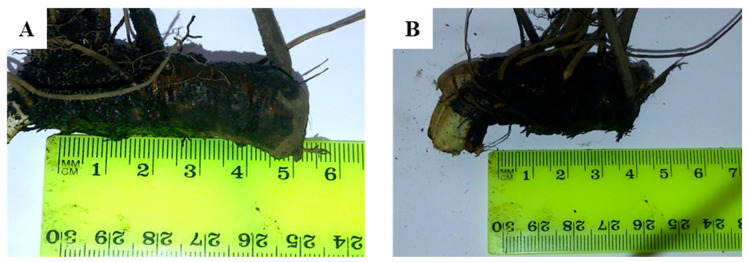
Different lengths of rhizome stocks of *A. amatymbica*, (**A**) 5 cm rhizome stock (**B**) 4 cm rhizome stock.

The foliage was cut off at the crown and rhizomes were washed under running tap water to remove adhering soil particles. Rhizomes were cut with a scalpel into six fragment lengths of 1, 2, 3, 4, 5 and 6 cm. A ruler was used to measure each fragment length (Figure 1) of *A. amatymbica*. Each fragment was weighed and tagged with different coloured plastic tapes to indicate the length of the rhizome and the weight before planting. The rhizome fragments used in this experiment were then wrapped in moist sterile tissue paper, placed in plastic bags, and taken to the greenhouse.

### 2.3. Design of the Experiment

A six by three factorial, randomised complete block design replicated three times was used in this study. The variables were the length of the rhizome and the planting depth. Three plots of different sowing depth (2.5 cm, 5 cm and 7.5 cm) and treatments (1, 2, 3, 4, 5 and 6 cm) were randomly assigned within the treatments. A total of 18 rhizome length fragments (1, 2, 3, 4, 5 and 6 cm) were planted at a depth of 2.5, 5 and 7.5 cm in each pot.

### 2.4. Planting of the A. amatymbica Fragments

The different fragment lengths of the rhizome (1, 2, 3, 4, 5, and 6 cm) were planted individually in plastic bags of 14 cm in diameter. Growth medium was added to the bottom of the plastic bag, and the depth was measured from the top of the plastic bag, using a ruler to ensure a correct planting depth. The fragments were then placed at the appropriate depth of 2.5, 5 and 7.5 cm and then topped up with soil. Each fragment was horizontally placed in the centre of each hole. The green house was maintained at 18–26 °C and 60% relative humidity. All plants were irrigated daily with tap water.

### 2.5. Shoot Emergence

**Figure 2:**
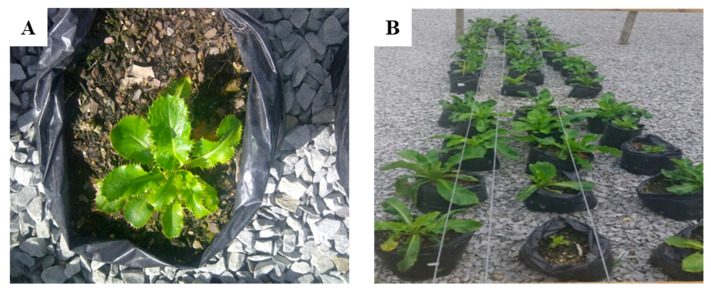
Cultivation of *A. amatymbica* (**A**) Shoots emerging from rhizome (**B**) Plants raised through rhizome segments growing in the greenhouse.

The emergence of leaves was recorded three times per week (2-day intervals) for 12 weeks (Figure 2). A plant was considered to have emerged if its sprouting shoot from the original fragment exceeded 1 cm in length. Cumulative emergence rate was calculated using the method of Pan et al. [30] using relation where emergence rate of leaves was regressed as a function of days after planting (DAP) using a logistic regression model:

(1) $$\mathrm{ER}\;\%=\frac{\Sigma \left(\mathrm{Number}\;\mathrm{of}\;\mathrm{leaves}\;\mathrm{emerged}\right)\left(\mathrm{DAP}\right)}{\mathrm{Total}\;\mathrm{number}\;\mathrm{of}\;\mathrm{leaves}\;\mathrm{emerged}}\times 100\%$$

where ER = *A. amatymbica* emergence rate (%); DAP = days after planting.

### 2.6. Leaf Area

Leaf area was determined by calculating

LA = 0.75 (length × width)(2)

where 0.75 is a constant [31].

### 2.7. Plant Height and Number of Leaves

A meter ruler was used to measure the shortest distance between the upper boundary of the main photosynthetic tissue on the plant and soil level [32]. Leaves formed were manually counted from each rhizome fragment length (*n* = 6) and its three replicates, and the mean number of leaves for each depth (*n* = 3) was determined.

### 2.8. Flower Yield

The number of florets per plant was counted and averaged for each treatment.

### 2.9. Survival, Shoot and Rhizome Moisture Content

The initial and final weight of the rhizomes were used to determine whether or not the fragment was alive. If the fragment gained weight, then it was considered alive. If the plant did not have any shoot and the rhizome had either lost weight or had decomposed entirely, then it was considered dead. The average weight of three replicates per rhizome fragment (*n* = 6) in each planting depth (*n* = 3) at the start and end of the experiment was determined for both nonregenerative and live fragments.

At the end of 30 weeks, plants were removed from the plastic bag, washed with tap water and separated into leaves and rhizomes. The fresh weight and the dry weight of the shoots and rhizome were determined by weighing the fresh plant material after which it was oven dried at 70 °C to a constant weight. Moisture content was expressed in percentage based on fresh weight using the equation below [33].

(3) $$\mathrm{Moisture}\;\mathrm{content}\;\left(\%\right)=\left(\frac{\mathrm{Fresh}\;\mathrm{weight}\;-\;\mathrm{dry}\;\mathrm{weight}}{\mathrm{Fresh}\;\mathrm{Weight}}\right)\;\times \;100\%$$

### 2.10. Data Analysis

Where applicable, data were subjected to the analysis of variance (ANOVA) using SAS (Statistical Analysis System) package. One way ANOVA in SAS was used to compare various growth parameters among treatments. A two-way ANOVA was also used to determine the interaction between planting depth and fragment length on various growth parameters. Mean separation test was conducted using Duncan’s multiple range tests.

## 3. Results

### 3.1. Effects of Planting Depth and Length of Rhizome on Shoot Emergence of A. amatymbica

**Figure 3:**
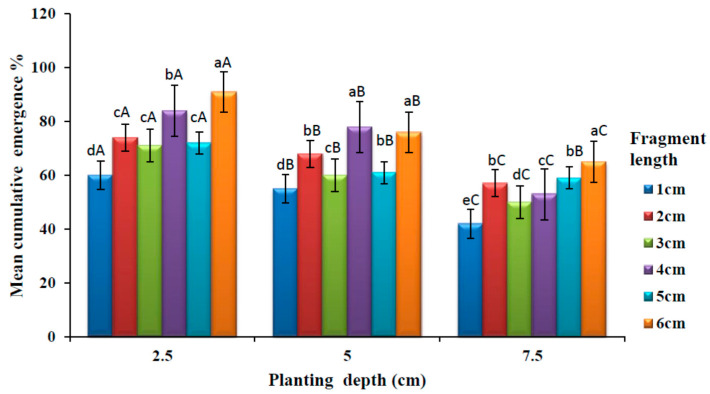
Emergence rate of *A. amatymbica* rhizome fragments of different lengths (1–6 cm) buried at different depths. Bars with different lowercase superscripts (a > b > c > d > e) in the same groups of planting depth are significantly different (*p* < 0.05) while bars with different uppercase superscripts (A > B > C) within a fragment length in the different groups of planting depth (2.5, 5 and 7.5 cm) are significantly different (*p* < 0.05).

The effects of planting depth and fragment length on shoot emergence are shown in Figure 3. It is observed that emergence decreased with increasing depth in all planting depth. There was a significant difference (*p* < 0.05) in cumulative shoot emergence among different rhizome lengths at 2.5 cm depth. There was no significant difference (*p* > 0.05) among the rhizome lengths 2, 3 and 5 cm. Six-centimetre fragment length produced higher cumulative shoot emergence. A significant difference in cumulative shoot emergence was observed (*p* < 0.05) among different fragment lengths at 5 cm planting depth. There was no significant difference (*p* > 0.05) between the rhizome length 4 and 6 cm. Similarly, there was also no significant difference (*p* > 0.05) with rhizome length 3 and 5 cm. The least cumulative shoot emergence was observed at 1 cm rhizome length. There was no significant difference (*p* > 0.05) on cumulative shoot emergence among different rhizome length at 7.5 cm planting depth. There was however, no significant difference (*p* > 0.05) on cumulative shoot emergence between rhizome length 2 and 5 cm. The highest cumulative emergence was observed at 6 cm rhizome length.

There was a significant difference (*p* < 0.05) in cumulative shoot emergence among different rhizome planting depth in all the rhizome fragment length. At 2.5 cm planting depth had significantly higher (*p* < 0.05) shoots emergence in all rhizome fragment lengths. There was no significant difference (*p* > 0.05) in cumulative shoots emergence between 5 and 7.5 cm planting depth at 5 cm rhizome length. The least cumulative shoot emergence was observed in 7.5 cm planting depth in all fragment lengths.

### 3.2. Effects of Planting Depth and Fragment Lengths of Rhizomes on the Number of Leaves Produced from Planting to Maturity

**Figure 4:**
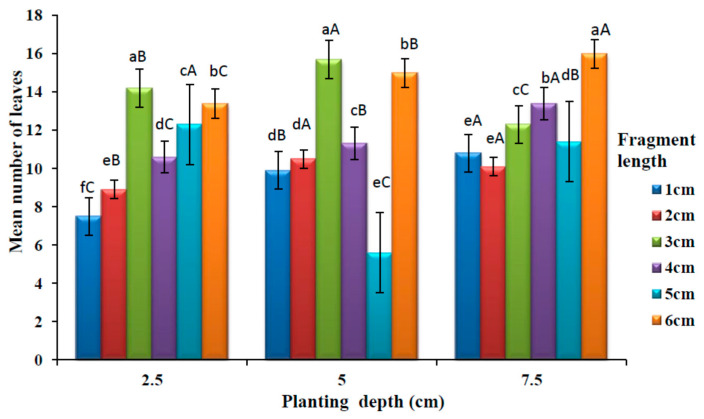
Effects of varying burial depths and fragments lengths of *A. amatymbica* rhizome on the number of leaves. Bars with different lowercase superscripts (a > b > c > d > e > f) in the same groups of planting depth are significantly different (*p* < 0.05) while bars with different uppercase superscripts (A > B > C) within a fragment length in the different groups of planting depth (2.5, 5 and 7.5 cm) are significantly different (*p* < 0.05).

The effects of planting depth and fragment length on the number of leaves are presented in Figure 4. There was a significant difference (*p* < 0.05) in the average number of leaves among different rhizome lengths at 2.5 cm planting depth. One centimetre rhizome produced the lowest number of leaves while the highest number of leaves were produced at 3 cm fragment length at a planting depth of 2.5 cm. A significant difference (*p* < 0.05) was observed in the mean number of leaves among different rhizome length at 5 cm planting depth. There was no significant difference (*p* > 0.05) between the rhizome lengths 1 and 2 cm. The highest number of leaves were observed at 3 cm rhizome length while the lowest was at 5 cm rhizome length. At 7.5 cm planting depth, a significant difference (*p* < 0.05) was observed in a mean number of leaves among different rhizome lengths. There was no significant difference (*p* > 0.05) between rhizome length 1 and 2 cm, which had the lowest number of leaves. The highest mean number of leaves was observed at 6 cm rhizome length.

There was a significant difference (*p* < 0.05) in the mean number of leaves among different rhizome planting depth for all the rhizome fragment lengths. No significant difference (*p* > 0.05) was observed on the mean number of leaves between the planting depths of 5 and 7.5 cm in 2 cm rhizome lengths. At a rhizome length of 6 cm, the highest number of leaves was observed at the deepest (7.5 cm) planting depth. On the other hand, the lowest number of leaves were obtained from the medium planting depth of 5 cm with 5 cm rhizome length.

### 3.3. Effects of Planting Depth and Fragment Lengths of A. amatymbica on Leaf Area

The effect of planting depth and rhizome length on the leaf area are shown in Table 1. There was a significant difference (*p* < 0.05) in average leaf area among different rhizome lengths at a planting depth of 2.5 cm. The rhizome lengths of 2 and 3 cm had lower average leaf area and were not significantly different (*p* > 0.05). Similarly, there was also no significant difference (*p* > 0.05) in leaf area between rhizome lengths 1 and 5 cm. The highest leaf area was observed at 4 cm rhizome length. There was a significant difference (*p* < 0.05) in average leaf area among different rhizome lengths at 5 cm depth. Lengths 2 and 3 cm showed no significant difference (*p* > 0.05), and the same trend was observed between length 4 and 5 cm, which had high average leaf area. The 2 cm rhizome length produced the lowest leaf area in the 5 cm depth. There was a significant difference (*p* < 0.05) in average leaf area among rhizome lengths at 7.5 cm depth, however, fragment length 1 and 6 cm were not significantly different (*p* > 0.05). Four-centimetre fragment length had significantly higher (*p* < 0.05) leaf area while 5 cm rhizome length had the lowest leaf area.

There was a significant difference (*p* < 0.05) in leaf area among different rhizome planting depths for all the rhizome fragment lengths. Shorter fragments lengths (1–3 cm) produced a higher leaf area at deeper planting (7.5 cm). At fragment length 4 and 6 cm, the highest leaf area was observed at a shallow planting depth (2.5 cm).

### 3.4. Effects of Planting Depths and Fragment Length on the Plant Height

**Figure 5:**
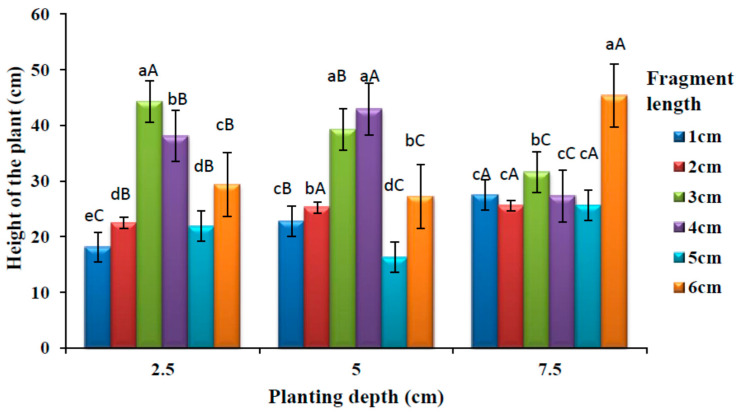
Effects of planting depths and rhizome lengths on the plant height. Bars with different lowercase superscripts (a > b > c > d > e) in the same groups of planting depth are significantly different (*p* < 0.05) while bars with different uppercase superscripts (A > B > C) within a fragment length in the different groups of planting depth (2.5, 5 and 7.5 cm) are significantly different (*p* < 0.05).

The effects of rhizome fragment length and burial depth on plant height is shown in Figure 5. There was a significant difference (*p* < 0.05) in the height of the plants among different rhizome lengths at 2.5 cm depth. No significant different (*p* > 0.05) was observed on the mean plant height between rhizome lengths 2 and 5 cm. The highest mean height was observed at 3 cm fragment length. There was a significant difference (*p* < 0.05) on the mean plant height among different rhizome lengths at 5 cm planting depth. There was no significant difference (*p* < 0.05) in plant height between the rhizome lengths 3 and 4 cm, which had a higher mean value of plant height than another fragment length. Similarly, there was also no significant difference (*p* > 0.05) in the mean plant height between 2 and 6 cm fragment lengths. The lowest mean plant height was observed in the 5 cm fragment length. There was a significant difference (*p* < 0.05) on the mean height among different rhizome length at 7.5 cm planting depth, and this difference was observed among the rhizome lengths 1, 2, 4 and 5 cm, which had lowest plant height values. A significantly higher (*p* < 0.05) mean height was observed at 6 cm fragment length.

A significant difference (*p* < 0.05) was observed in plant height among different rhizome planting depths for all the rhizome fragment lengths. Longer fragment length (6 cm) produced taller plants at deeper planting (7.5 cm). There was no significant difference (*p* > 0.05) in the plant height between 5 and 7.5 cm planting depth at 2 cm fragment length. The lowest plant height was produced at a medium planting depth (5 cm) at 5 cm rhizome length.

### 3.5. Effects of Planting Depths and Fragment Lengths on the Number of Florets

**Figure 6:**
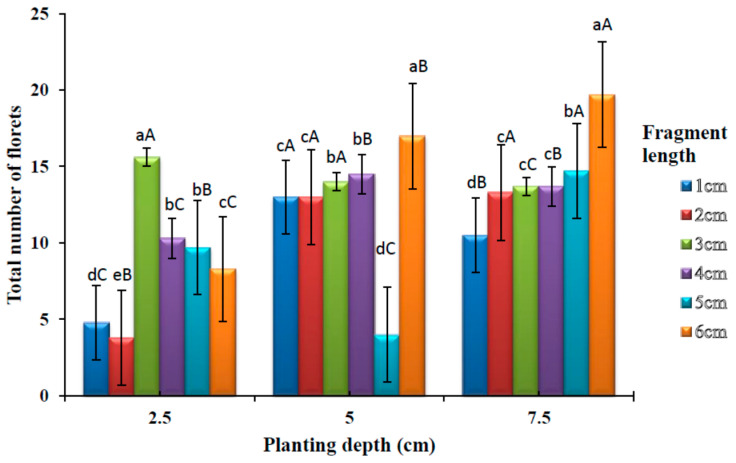
Effects of varying planting depths and rhizome fragment lengths on the number of florets. Bars with different lowercase superscripts(a > b > c > d > e) in the same groups of planting depth are significantly different (*p* < 0.05) while bars with different uppercase superscripts (A > B > C) within a fragment length in the different groups of planting depth (2.5, 5 and 7.5 cm) are significantly different (*p* < 0.05).

The cultivated *A. amatymbica* plants started flowering at 21 weeks after planting. The number of florets produced from varying rhizome lengths and planting depths are shown in Figure 6. There was a significant difference (*p* < 0.05) in the number of florets among different rhizome lengths at 2.5 cm depth. No significant difference (*p* > 0.05) was observed on the number of florets between 4 and 5 cm rhizome lengths. The highest number of florets was observed at 3 cm rhizome length. At 5 cm planting depth, there was a significant difference (*p* < 0.05) in the number of florets among different rhizome lengths, however rhizome lengths 3 and 4 cm did not show any significant difference (*p* > 0.05). Similarly, no significant difference (*p* > 0.05) was observed between rhizome lengths 1 and 2 cm. The highest number of florets was observed in the 6 cm fragment length. There was a significant difference (*p* < 0.05) in the number of florets among different rhizome lengths at 7.5 cm planting depth. There was, however, no significant difference (*p* > 0.05) among fragment lengths 2, 3 and 4 cm. The highest number of florets was observed in the 6 cm rhizome length.

There was a significant difference (*p* < 0.05) in a number of florets among different rhizome planting depths for all the rhizome fragment lengths. No significant difference was observed between 5 cm and 7.5 cm, planting depths at 2 cm fragment length. The highest number of florets was observed at deepest planting depth (7.5 cm) with 6 cm fragment length.

### 3.6. Effects of Planting Depths and Fragment Lengths Production on Rhizome Length Gain

The effects of planting depths and fragment lengths on rhizome length gain are presented in Table 2. There was a significant difference (*p* < 0.05) in rhizome length gain among different rhizome lengths at 2.5 cm planting depth, however, there was no significant difference (*p* > 0.05) between rhizome length 4 and 5 cm. The same trend was observed between rhizome lengths 2 and 3 cm. Six-centimetre rhizome length produced the lowest fragment length gain. A significant difference (*p* < 0.05) was observed in rhizome length gain among different rhizome lengths planted at 5 cm depth. However, there was no significant difference (*p* > 0.05) in the rhizome length gain produced between rhizome lengths 1 and 2 cm, which were longer than others. Similarly, there was also no significant difference (*p* > 0.05) in the rhizome length gain between rhizome lengths 4 and 6 cm. The lowest rhizome length gain was produced in the 5 cm length. There was a significant difference (*p* < 0.05) in the rhizome length gain of the rhizomes lengths planted at 7.5 cm depth. There was, however, no significant difference (*p* > 0.05) in rhizome length gain between rhizome lengths of 5 and 6 cm, which had the lowest length gain than others. Similarly, there was no significant difference (*p* > 0.05) in the rhizome length gain between rhizome lengths 3 and 4 cm. The longest final rhizome length was obtained from 1 cm rhizome length at 7.5 cm depth.

There was a significant difference (*p* < 0.05) in rhizome length gain among different planting depths for all the rhizome fragment lengths. One centimetre fragment length had the longest rhizome length gain in all burial depths. The longest rhizome length gain was observed in the 2 cm rhizome length at medium depth (5 cm).

### 3.7. Effects of Planting Depths and Fragment Lengths on Rhizome Weight Gain

The effects of planting depths and fragment lengths on rhizome weight gain are presented in Table 3. All fragment lengths and burial depths showed 100% survival. There was a significant difference (*p* < 0.05) in the rhizome weight gain among different rhizome lengths at 2.5 cm planting depth. However, there was no significant difference (*p* > 0.05) between rhizome lengths 4 and 6 cm, which were recorded the highest weight values. Similarly there was also no significant difference (*p* > 0.05) in the rhizome weight gain between 1 and 5 cm rhizome fragment lengths. The lowest rhizome weight gain was observed in the 2 cm fragment length at 2.5 cm planting depth. There was a significant difference (*p* < 0.05) in the rhizome weight gain among different rhizome lengths at 5 cm planting depth. The highest rhizome weight gain was obtained from 6 cm rhizome lengths while the lowest was obtained from 4 cm rhizome length. There was a significant difference (*p* < 0.05) in the rhizome weight gain among different rhizome lengths at 7.5 cm planting depth. The highest rhizome weight gain was produced from 6 cm fragment length while 1cm fragment length produced the lowest rhizome weight gain.

At all burial depths, there was a significant difference (*p* < 0.05) in rhizome weight gain in all rhizome fragment lengths. Shorter fragment length (1–3 cm) produced the highest rhizome weight gain at 5 cm planting depth compared to 2.5 cm and 7.5 cm plamting depth. Suprisingly the highest rhizome weight gain was produced at 5 cm planting depth by 6 cm long rhizomes. The lowest rhizome weight gain was also produced at 5 cm planting depth with 4 cm rhizomes fragment length.

### 3.8. Effects of Rhizome Length and Planting Depth on the Moisture Content of Rhizome

The harvest rhizome moisture content for different rhizome lengths and planting depths are presented in Table 4. There was a significant difference (*p* < 0.05) in moisture content among different rhizome fragment lengths at 2.5 cm planting depth. There was however, no significant difference (*p* > 0.05) in moisture content between rhizome lengths 2 and 5 cm. A similar trend was observed between fragment lengths 3 and 4 cm, which had lower moisture content. There was also no significant difference (*p* > 0.05) in moisture content between 1 and 6 cm rhizome lengths, which had higher moisture content values than others. There was a significant difference (*p* < 0.05) in moisture content among different rhizome lengths at 5 cm planting depth. There was however, no significant difference (*p* > 0.05) among rhizome lengths 2, 4, 5 and 6 cm. The 3 cm fragment length had the highest moisture content. There was a significant difference (*p* < 0.05) in moisture content among different rhizome lengths at 7.5 cm planting depth. Non-significant differences (*p* > 0.05) were, however, observed in the rhizome moisture content among rhizome lengths 1, 2, 3, and 4 cm, which recorded lower moisture content values. There was no significant difference (*p* > 0.05) in moisture between rhizome lengths 5 and 6 cm. Higher moisture content.was observed in 5cm fragment length.

At all burial depths, there was no significant difference (*p* > 0.05) in the rhizome moisture content of different rhizome fragment lengths. There was no significant difference (*p* > 0.05) in the rhizome moisture content in all burial depth between rhizome lengths 2 and 6 cm. One-centimetre rhizome length had the highest rhizome moisture content in the shallow burial depth (2.5 cm).

### 3.9. Effects of Rhizome Lengths and Planting Depths on the Shoot Moisture Content

The rhizome shoot moisture content at different rhizome lengths, and planting depths are presented in Table 5. There was a significant difference (*p* < 0.05) in shoot moisture content among different rhizome lengths at 2.5 cm planting depth. Shoot moisture content among rhizome lengths 3, 4, and 5 cm was, however, not significantly different. The highest shoot moisture content was obtained from 6 cm rhizome length. A significant difference (*p* < 0.05) was observed in shoot moisture content among different rhizome lengths at 5 cm planting depth. There was however, no significant difference (*p* > 0.05) in shoot moisture content among rhizome lengths 2, 3 and 5 cm. Similarly, there was no significant difference (*p* > 0.05) between rhizome length 4 and 6 cm. The lowest moisture content was obtained from the 1 cm fragment length. Shoot moisture content significantly differed (*p* > 0.05) among different rhizome lengths at 7.5 cm planting depth. However, shoot moisture content among rhizome lengths 1, 2, 3, and 4 cm were not significantly different and recorded the lowest moisture contents. The highest moisture content was obtained from the 5 and 6 cm rhizome lengths.

At all burial depths, there was a significant difference among shoot moisture content of different rhizome fragment lengths. Shoot moisture content was the highest at 2.5 cm depth for all rhizome fragment lengths. The lowest shoot moisture was observed in 5 cm planting depth with 1 cm fragment length.

## 4. Discussion

Increase in planting depth significantly reduced the emergence rate of *A*. *amatymbica*. At 2.5 cm depth, the emergence was found to be higher than at 5 and 7.5 cm depths regardless of rhizome fragment lengths. Previous studies on *Alternanthera philxeroides* (Mart) Standl. and *Physalis viscosa* L. also reported that deeper burial depth decreased the rate of emergence of shoots [34,35]. This finding was also in agreement with Soltani et al., [36] who showed that shoots had higher percentage emergence in shallow planting depths. Thus, it is clear that planting depth is one of the significant factors that influence the rate of emergence. Rask and Andreasen [37] showed that planting depth was related to delay in emergence in *Mentha arvensis* (L) [38] and *Calystegia sepium* (L.) R.Br. [22]. One possible reason is that planting depth has been the major environmental stress on the emergence of shoots since it could significantly change abiotic conditions such as temperature, active photosynthetic radiation, moisture and soil organic matter [39,40]. The survival and growth of rhizome fragments buried in the deeper soils may mostly rely on the utilisation of reserves stored in the rhizomes when carbohydrates cannot be provided through photosynthesis [41]. It has been reported that rhizome buried in the deeper soils may deplete all stored carbohydrates before new shoots reach the soil surface which will increase the risk of plant regeneration [28].

The current study showed that longer rhizome fragments increased shoot emergence rate. These results are consistent with a previous study [42]. Other authors have suggested that longer rhizome clonal fragments have more carbohydrate reserves in the rhizome [43] and that plants originating from short fragments were less competitive than those from long fragments [44]. It has been reported that longer rhizome length may facilitate the survival and growth of clonal fragments length because rhizome length may be positively correlated with the amount of stored carbohydrates [45]. This suggests that food reserves stored in the longer rhizomes can be remobilised and reused to increase plant growth [27].

The present study showed that planting depth and rhizome fragment length influenced the growth and development of leaves, as it was observed that an increase in planting depth increased the number of leaves. Generally, 5 cm and 7.5 cm planting depth resulted in more leaves than 2.5 cm depth. These results suggest that there may be some stimulating effects of planting depth and fragment length on plant vigour. It has been reported that planting depth affects better utilisation of nutrients and greater ability to respond to favourable conditions which will result in more vigorous plant growth [46]. Similar results have been reported for soybean [30]. Number of leaves produced in the longer rhizome may be a result of rhizome length being positively correlated to levels of carbohydrates, proteins and other nutrients [28]. This study was in agreement with Weber [47], who found that fragment length plays an important role in shoot formation.

In the present study, it was observed that 1, 2, 5, and 6 cm long rhizomes planted at 7.5 cm depth produced the tallest plants. This result could be explained by the fact that plants sowed at shallow depths often do not grow as vigorously as those sowed deeper [48]. There is evidence that moderate burial can improve the photosynthetic capacity and vigour of buried plants after emergence, leading them to compensate for the consumption of storage reserves caused by burial [49]. This could possibly explain why *A. amatymbica* plants at 7.5 cm depth had the highest number of leaves, which compensated for the delayed emergence rate. This was also reported by Monaco et al. [50] that as the fragments survive the cultivation from deep planting depth, it can quickly respond and accumulate dry matter, repeated cultivation would be necessary to exhaust stored carbohydrates and improve crop productivity. This could suggest that the rhizome could be reused for cultivation and this would reduce overharvesting of *A. amatymbica*.

The plants originating from short fragments in all planting depths showed reduced growth performace, while long fragments resulted in higher plant productivity. This agrees with previous findings that suggested that non-structural carbohydrates and soluble proteins stored in the rhizome can be reutilised for the regeneration of plants that are subjected to disturbance [51,52].

Leaf area is an important variable for most ecophysiological studies in terrestrial ecosystems concerning light interception, evapotranspiration, and photosynthetic efficiency in plant growth [53]. The growth of the leaf is important because it is valuable for plant nutrition, competition, soil water relations, and protection measures [54]. The life cycle of a plant usually involves the early phase of increasing photosynthetic rate while the leaf expands. In the present study, planting depth did affect leaf area. The results indicated that leaf area at 7.5 cm planting depth, from 1–3 cm rhizomes were higher compared to other burial depths. The reduction of leaf area in other burial depths may be caused by the influence of shallow depth, which then influences the water content. This results in the reduction of stem length and leaf area of the plant so as to adapt to the amount of light or intercepted light, which affects the final product [55]. It was observed that 2.5 cm planting depth had a higher number of leaves at emergence, and this affected the leaf area. This might be due to the nutrients competition of leaves populated in one plant [56]. The population on the number of leaves restricts transpiration including the closure of the stomata and less water evaporating from the leaf surface. Further, it will restrict the efficiency of the photosynthesis process and limit crop productivity [41]

Flower initiation marks an important transition from vegetative development to reproductive development, which is the most crucial event in the life cycle of a plant. The flower produces the reproductive cells of plant and seeds for the next generation. The seeds enable the plant embryos to survive before seedlings establishment, thereby ensuring the initiation of the next generation of plants [57]. Plant depth had a crucial effect on flower production by *A. amatymbica*. Plants buried at depths of 5 cm and 7.5 cm produced more flowers than those planted at a shallower depth (2.5 cm). Similar findings were reported by De Hertogh and Lenard [58]. The highest number of flowers produced at 5 cm and 7.5 cm depth may be related to the fact that the highest number of leaves were produced in those fragment lengths. Increase in fragment length also had an effect on the number of florets produced, which might be due to the fact that flowering and flower production, are dependent on vegetative growth and sufficient supply of essential nutrients by longer rhizomes [59,60]. The fact that in all fragment lengths and depths of *A. amatymbica* formed flowers is an indication that rhizome cultivation will be a useful conservation method or approach for this plant.

Planting depth and fragment length had effects on the production of the rhizome biomass. The shorter fragments (1–3 cm) in this study had a higher yield on the rhizome weight at a planting depth of 5 cm. Similar findings were also obtained by Anbari et al. [61] who reported that fragmentation into short pieces requires less stored energy to initiate the rhizome growth which makes short fragments to have more vigorous growth than longer fragments. The present study indicated that short fragments generally produced the longest rhizomes. This was also in line with Adreasen and Rask [37] that fragment length has an impact on the biomass of the rhizome. The decrease in rhizome biomass may be caused by the number of leaves which may affect the levels of assimilates being accumulated by plants due to reduced photosynthesis. The 5 cm planting depth in all fragment length would be the recommended propagation regime due to the highest rhizome weight produced. This study indicated that cultivation of *A. amatymbica* could reduce pressure on the wild population as a means of conserving this vulnerable medicinal plant and to address the demand for traditional medicine.

The highest fresh rhizome weights observed in this study may be attributed to the development in plant height and a number of leaves which influence rhizome biomass. This study also validated the work of Asaeda and Nam [62]. The differences in above to below ground biomasses in Table 3 could be associated with higher sprouting efficiency. At all planting depths, shoot fresh weight was lower than rhizome fresh weight while shoot moisture content was higher than that of the rhizome [63]. The moisture content of the shoots ranged from 50% to 68% in all fragments and all burial depths. Water is one of the most important requirements in plant life and production, as the absence of water will affect transpiration including the closing of stomata which will automatically reduce the photosynthetic rate and limit plant growth and productivity [41].

## 5. Conclusions

The results of the study indicated that planting depths and fragment lengths of *A. amatymbica* cannot prevent its survival. Although sowing depth had an impact on the emergence of leaves *A. amatymbica* managed to recover from delay in the emergence, and responded positively to the planting stress. Shorter fragments at all burial depth of 5 cm produced the highest rhizome length gain. Medium planting depth (5 cm) is recommended for cultivation of *A. amatymbica* as it produces highest rhizome biomass required by traders, herbalists, and traditional healers. These results can play an important role in the establishment of propagation protocols of *A. amatymbica*. Vegetative propagation using rhizome could help to meet the current and future demands on *A. amatymbica*.

## Figures and Tables

**Table 1:** Effects of planting depths and rhizome fragment lengths on the leaf area of *A. amatymbica*.

Rhizome Fragment Length (cm)	Planting Depth (cm)		
	2.5 cm	5 cm	7.5 cm
	Leaf Area (cm^2^)		
1 cm	87.34 ± 4.1 ^c B^	70.92 ± 5.8 ^d C^	91.30 ± 3.4 ^d A^
2 cm	74.51 ± 5.1 ^d B^	94.52 ± 4.1 ^b A^	97.96 ± 1.6 ^c A^
3 cm	77.08 ± 3.8 ^d C^	92.92 ± 2.7 ^b B^	100.8 ± 1.7 ^b A^
4 cm	111.9 ± 9.2 ^a A^	101.3 ± 3.4 ^a C^	105 ± 5.8 ^a B^
5 cm	92.21 ± 1.8 ^c B^	98.36 ± 1.7 ^a A^	86.38 ± 3.1 ^e C^
6 cm	103 ± 4.3 ^b A^	84.11 ± 4.1 ^c C^	91.53 ± 3.9 ^d B^

Means with different uppercase superscripts (A > B > C) in the same rows are significantly different (*p* < 0.05) while means with different lowercase superscripts(a > b > c > d > e) within a column are significantly different (*p* < 0.05) Values are mean ± SD (*n* = 3).

**Table 2:** Effects of planting depths and fragment lengths production on the rhizome length gain.

Initial Rhizome Fragment Length (cm)	Planting Depth (cm)		
	2.5 cm	5 cm	7.5 cm
	Rhizome Length Gain (cm)		
1 cm	7.03 ± 0.35 ^a B^	8.03 ± 0.2 ^a A^	8.06 ± 0.54 ^a A^
2 cm	4.7 ± 0.84 ^b C^	8.5 ± 0.74 ^a A^	6.9 ± 1.7 ^c B^
3 cm	4.4 ± 0.26 ^b C^	6.6 ± 0.17 ^b B^	7.2 ± 0.58 ^b A^
4 cm	5.4 ± 0.29 ^c B^	4.0 ± 0.42 ^c C^	7.2 ± 0.95 ^b A^
5 cm	5.1 ± 0.73 ^c A^	3.5 ± 1.10 ^d B^	5.6 ± 0.42 ^d A^
6 cm	3.2 ± 1.15 ^d C^	4.3 ± 0.78 ^c B^	5.9 ± 0.61 ^d A^

Means with different uppercase superscripts (A > B > C) in the same row are significantly different (*p* < 0.05), while means with different lowercase superscripts (a > b > c > d) within a column are significantly different (*p* < 0.05). Values are mean ± SD (*n* = 3).

**Table 3:** Effects of planting depth and fragment length on the rhizome weight gain.

Rhizome Fragment Length (cm)	Planting Depth (cm)		
	2.5 cm	5 cm	7.5 cm
	Rhizome Weight Gain (g)		
1 cm	140 ± 3.1 ^c B^	208 ± 6.9 ^d A^	134 ± 4.3 ^f C^
2 cm	128 ± 6.1 ^d C^	252 ± 9.07 ^c A^	177 ± 4.01 ^d B^
3 cm	182 ± 7.6 ^b C^	246 ± 12.1 ^b A^	201 ± 3.1 ^c B^
4 cm	219 ± 13.4 ^a A^	99.8 ± 3.4 ^f B^	217 ± 13.9 ^b A^
5 cm	143 ± 6.9 ^c C^	157 ± 5.79 ^e B^	171 ± 7.4 ^e A^
6 cm	221 ± 7.3 ^a C^	297 ± 2.74 ^a A^	230 ± 8.8 ^a B^

Means with different uppercase superscripts (A > B > C) in the same row are significantly different (*p* < 0.05), while means with different lowercase superscripts (a > b > c > d > e > f) within a column are significantly different (*p* < 0.05). Values are mean ± SD (*n* = 3).

**Table 4:** Effect of planting depth and fragment length on the rhizome moisture content of *A. amatymbica*.

Rhizome Fragment Length (cm)	Planting Depth (cm)		
	2.5 cm	5 cm	7.5 cm
	Rhizome Moisture (%)		
1 cm	57.3 ± 1.8 ^a A^	49.2 ± 2.8 ^c B^	53.4 ± 3.3 ^b B^
2 cm	51.2 ± 1.09 ^b A^	51.2 ± 1.89 ^b A^	52.53 ± 1.20 ^b A^
3 cm	45.5 ± 1.26 ^c B^	54.1 ± 1.26 ^a A^	52.61 ± 2.56 ^b A^
4 cm	48.8 ± 1.3 ^c B^	52.4 ± 1.6 ^b A^	51.8 ± 1.45 ^b A^
5 cm	50.5 ± 0.3 ^b B^	51.5 ± 2.84 ^b B^	56.5 ± 2.01 ^a A^
6 cm	54.5 ± 2.3 ^a A^	53.3 ± 2.7 ^b A^	54.6 ± 1.4 ^a A^

Means with different uppercase superscripts (A > B) in the same row are significantly different (*p* < 0.05), while means with different lowercase superscripts (a > b > c) within a column are significantly different (*p* < 0.05). Values are mean ± SD (*n* = 3).

**Table 5:** Effect of planting depth and fragment length on the shoot moisture content of cultivated *A. amatymbica*.

Rhizome Fragment Length (cm)	Planting Depth (cm)		
	2.5 cm	5 cm	7.5 cm
	Shoot Moisture (%)		
1 cm	50.8 ± 1.2 ^d A^	38.71 ± 2.3 ^c B^	47.9 ± 1.4 ^b A^
2 cm	57.8 ± 2.2 ^c A^	54.2 ± 0.6 ^a A^	50.4 ± 1.72 ^b B^
3 cm	63.2 ± 2.1 ^b A^	56.03 ± 1.2 ^a B^	47.6 ± 2.5 ^b C^
4 cm	60.7 ± 1.64 ^b A^	49.28 ± 2.33 ^b B^	50.7 ± 1.66 ^b B^
5 cm	61.83 ± 1.82 ^b A^	53.22 ± 2.08 ^a C^	57.6 ± 1.31 ^a B^
6 cm	68.23 ± 2.5 ^a A^	49.78 ± 2.3 ^b C^	55.9 ± 2.6 ^a B^

Means with different uppercase superscripts (A > B > C) in the same rows are significantly different (*p* < 0.05) while means with different lowercase superscripts(a > b > c > d) within a column are significantly different (*p* < 0.05). Values are mean ± SD (*n* = 3).
